# HIV Prevalence, Virological Suppression, and Consistent Condom Use Among Social Venue-Going Men in Zimbabwe: Insights from the 2022 Priorities for Local AIDS Control Efforts (PLACE) Surveys

**DOI:** 10.1007/s10461-026-05084-8

**Published:** 2026-03-14

**Authors:** Galven Maringwa, Sungai T. Chabata, Fortunate Machingura, Jaspar Maguma, Memory Makamba, Tariro Chinozvina, Samson Chikura, Leslie Nyoni, Madonna Mlambo, Edward Matsikire, Amon Mpofu, Raymond Yekeye, Benard Madzima, Owen Mugurungi, Brian Rice, Sharon Weir, James R. Hargreaves, Elizabeth Fearon, Frances M. Cowan

**Affiliations:** 1 Centre for Sexual Health, HIV/AIDS Research Zimbabwe (CeSHHAR Zimbabwe), 10 Coronation Road, Greendale, Harare, Zimbabwe; 2 Department of International Public Health, Liverpool School of Tropical Medicine, Liverpool, UK; 3 Department of Social and Environmental Health Research, Public Health and Policy, London School of Hygiene and Tropical Medicine, London, UK; 4 Institute for Global Health, University College London, London, UK; 5 National AIDS Council, Harare, Zimbabwe; 6 AIDS & TB Unit, Ministry of Health and Child Care, Harare, Zimbabwe; 7 Department of Epidemiology, The Gillings School of Global Public Health, The University of North Carolina at Chapel Hill, Chapel Hill, NC USA; 8 School of Medicine, and Population Health, University of Sheffield, Sheffield, UK

**Keywords:** HIV prevalence, Virological suppression, Consistent condom use, Social venue-going men, Paying for sex, Targeted interventions

## Abstract

Men attending social venues face barriers to accessing HIV prevention and care services. These venues—such as bars, guesthouses, nightclubs, and transport hubs—facilitate new sexual partnerships but lack cohesive social networks, making it challenging to design and implement effective HIV prevention strategies. Men who attend social venues are more likely to pay for sex, potentially increasing their risk of acquiring or transmitting HIV. However, data on how HIV-related behaviours and service engagement differ between men who do and do not pay for sex among those attending venues remain limited. This study examines whether men who pay for sex have higher rates of HIV prevalence, lower rates of virological suppression, and poor HIV-prevention-related behaviours compared to those who do not. Using the Priorities for Local AIDS Control Efforts (PLACE) methodology, we collected cross-sectional data from April to December 2022 across 190 venues in four cities and towns in Zimbabwe. Participants underwent finger-prick HIV testing; those testing positive provided dried blood spots (DBS) for viral load measurement. We also collected sexual behaviour data, including condom use. We applied survey weights and used weighted Poisson regression models with robust standard errors to investigate factors associated with HIV status, virological suppression, and condom use among venue-going men, treating paying for sex as the primary exposure variable. All reported percentages are weighted. Among venue-going 2,827 men, 984 (40.1%) reported paying for sex in the past 12 months, and 531 (15.1%) reported consistent condom use in the past month. Overall, HIV prevalence was 10.7%. Among men living with HIV, virological suppression was 67.9%. In adjusted analyses, there were no significant associations between paying for sex and HIV status (adjusted prevalence ratio (aPR) = 1.12, 95% CI: 0.64–1.94), self-reported consistent condom use in the past month (aPR = 0.87, 95% CI: 0.57–1.34), or rates of virological suppression among men living with HIV (aPR = 0.97, 95% CI: 0.71–1.34). Findings indicate substantial HIV risk and suboptimal prevention and treatment engagement among men frequenting social venues, irrespective of paying for sex. Therefore, targeted interventions are needed for both paying and non-paying men.

## Introduction

Though there has been broad progress in coverage of HIV treatment and expanded access to prevention services, HIV/AIDS remains a serious global health problem, particularly in sub-Saharan Africa, which harbours nearly two-thirds (65.0%) of all infections [1]. Men who patronize venues and pay for sex are at increased risk for HIV and STIs due to a combination of factors, including inconsistent condom use within commercial transactions, low rates of recent HIV testing, and engagement in high-frequency or concurrent sexual partnerships [2, 3]. According to Zimbabwe’s 2015 Demographic and Health Survey (ZDHS), 4.0% of men aged 15–54 reported paying for sex in the past 12 months [4]. In 2022, clients of FSWs accounted for an estimated 10.0% of new HIV diagnoses globally [1].

Despite extensive research on HIV epidemiology and service uptake among female sex workers (FSWs), data on men who have sex with FSWs remain limited. This gap includes information about their patterns of condom use, pre-exposure prophylaxis (PrEP) uptake, voluntary medical male circumcision (VMMC) status if HIV negative, and engagement with treatment if living with HIV. Although effective treatment adherence leads to virological suppression and prevents sexual transmission [5], men generally have poorer outcomes than women across the HIV care continuum, from HIV status awareness to optimal use of ART and virological suppression, and are therefore characterised as ‘left behind’ in HIV response efforts [6].

As a result, these men are often underserved in terms of HIV services because they are difficult to target as a coherent population, contributing to higher rates of HIV transmission. Social venue-going men exhibit high-risk behaviours associated with HIV [7–9]. Among men recruited at social venues in South Africa, those who were 25 years or older, had not completed high school, did not use condoms at first sex, consumed alcohol or substances, or were not medically circumcised were more likely to have a positive HIV status compared to men without these characteristics [10]. Social venue-going men also exhibit low uptake of HIV prevention services, including consistent condom use [9]. Consistent condom use offers dual protection against both STIs, including HIV, and unwanted pregnancies, and it is highly effective [11, 12]. Condom use with heterosexual partners is influenced by a range of socio-demographic, behavioural, psychosocial, relationship, contextual, and structural factors [13]. Factors associated with inconsistent condom use include age at sex debut, illicit drug use [14], marital status or partner type [15–20], and perceived high self-efficacy for condom use [21].

To better inform more targeted prevention and treatment programming for men at high risk, this study assessed prevalence of HIV, virological suppression, and HIV-related risk behaviours among men attending social venues in four Zimbabwean cities and towns. We sought to: [1] determine the overall burden of HIV and gaps in the treatment cascade; and [2] compare HIV prevalence, risk behaviours, and effective treatment coverage as determined by virological suppression between men reporting paying for sex in the past 12 months and those not reporting these behaviours.

## Methods

### Study Design and Population

**Fig. 1:**
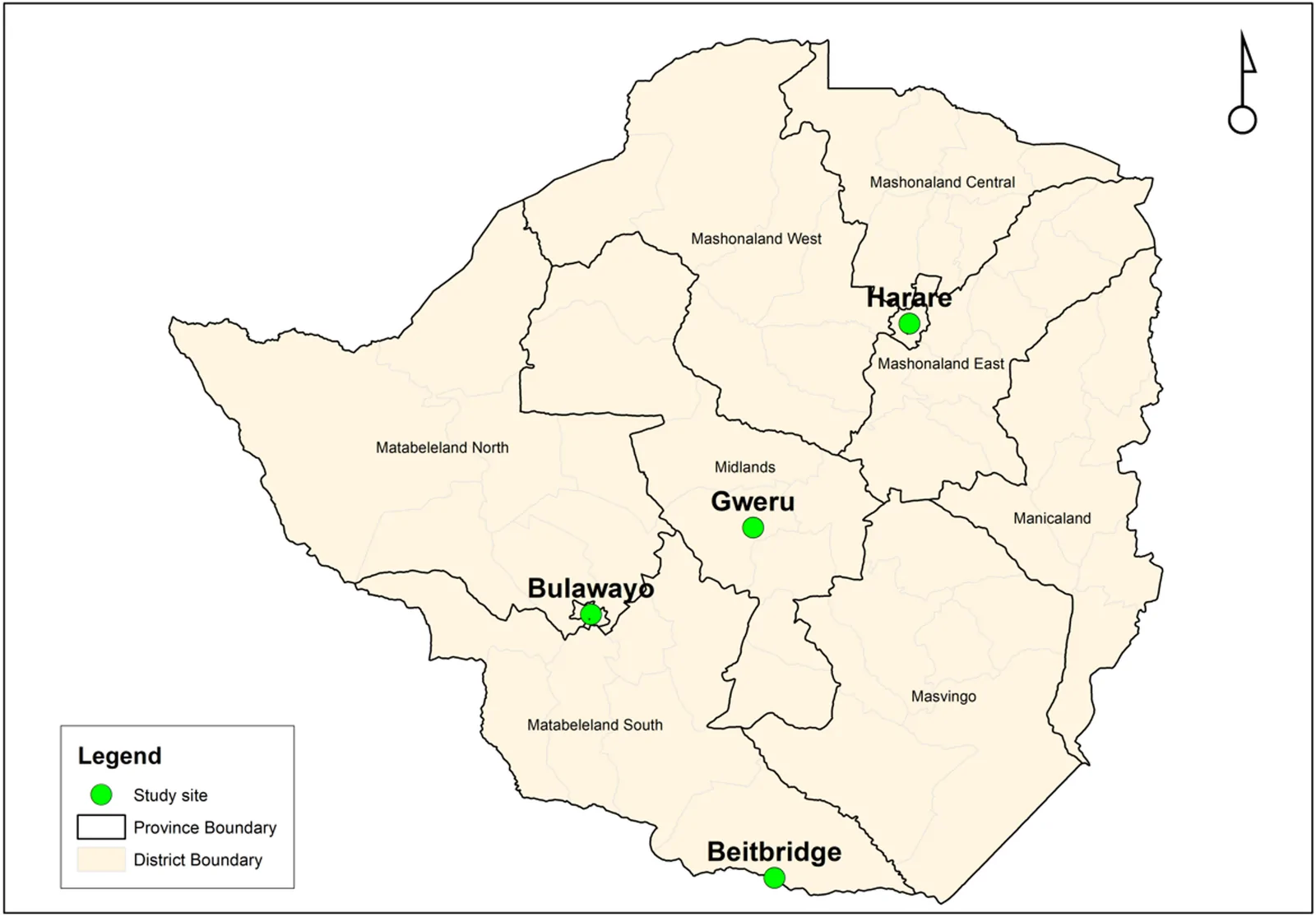
Map of Zimbabwe showing PLACE study cities and towns

Between April and December 2022, we applied the Priorities for Local AIDS Control Effort (PLACE) methodology to collect data from venue patrons and workers in four cities and towns across four different provinces in Zimbabwe (Fig. 1). The Ministry of Health and Child Care (MoHCC) and National AIDS Council (NAC) selected provinces for the surveys based on existing epidemiological data and contextual information. PLACE aims to generate detailed, locally relevant data to guide HIV prevention and care by mapping social venues where individuals meet new sexual partners, using a systematic, multi-phase approach that includes stakeholder engagement, community informant surveys, venue informant surveys, and biobehavioural surveys (BBS) [22–30]. In Phase 1, we worked with stakeholders (e.g., provincial medical directors, local health officials, HIV response partners, community advisory boards, monitoring and evaluation directors, and the National AIDS Council) to identify Priority Prevention Areas (PPAs). Priority Prevention Areas are geographic locations, venues, or subpopulations where the risk of HIV transmission and acquisition is highest, and where targeted interventions can have the greatest population-level impact by reducing new infections and improving engagement in care. In Phase 2, we generated lists of social venues (e.g., bars, guesthouses, nightclubs, and transportation hubs) by asking knowledgeable community informants (e.g., bartenders, police, taxi drivers, street vendors, and peer educators) to name all the venues they knew (community informant survey). In Phase 3, we visited all previously identified venues and classified them as operational, not found, temporarily closed, permanently closed, or duplicates (venue informant survey). Operational venues were profiled for operating hours and busy times, activities and amenities, the availability of on-site prevention services such as HIV testing, peer education, condom distribution, and estimated attendee populations.

Finally, in Phase 4, we conducted the BBS at a random sample of these venues on busy nights, interviewing patrons and staff and offering HIV testing. A biobehavioural survey (BBS) is a standardized, cross-sectional research method that concurrently collects and links biological data (e.g., HIV/STI prevalence, viral load) with behavioural and demographic data (e.g., sexual practices, service utilization, substance use) from the same participants. This integrated design is a cornerstone of surveillance for key populations, as it enables the direct analysis of how specific behaviours and social contexts interface with biological outcomes, thereby elucidating transmission dynamics and gaps in prevention and care services within defined communities or risk groups [31, 32].

In Bulawayo, Zimbabwe’s second-largest city, stakeholders purposively chose one of three Priority Prevention Areas because, unlike other Priority Prevention Areas, it encompassed each identified venue type and the Central Business District. Among 198 venues identified in this Priority Prevention Area, 52 were randomly selected via stratified sampling. In Gweru, a mid-sized city, 190 venues were identified and 30 were randomly selected for the BBS. In Beitbridge, a key border town adjacent to South Africa, characterised by significant cross-border traffic, 143 venues were identified and 30 were selected for the BBS. In Harare, the capital and largest city, 2,456 venues were mapped across 196 districts, and three districts were purposively selected for the BBS based on District Technical Working Group recommendations. From these districts, 78 venues were chosen through stratified random sampling.

### Data Collection

At each selected venue, BBS participants included male and female patrons and workers aged 16 years or older who were present during the evening hours on Friday, Saturday, and Sunday. We recruited all workers in venues with fewer than 10 workers. Venue quotas of individuals interviewed and tested for HIV were pre-calculated proportionately to expected venue attendance (venue informant surveys). In each venue, enumerators counted and calculated the sampling interval (n) during peak times (total venue patrons ÷ quota) and systematically sampled every nth eligible man from random starting points until reaching quotas. All on-site participants provided informed consent by marking an ‘X’ on the consent form, while witnesses signed the forms. Participants then completed an interviewer-administered questionnaire. No data on refusals was collected. Individual refusal tracking, though feasible, was erroneously omitted during rapid anonymous data collection from mobile venue populations.

The questionnaire was administered in the predominant local languages of the regions, by trained, matched interviewers (i.e., Shona and Ndebele). All materials were translated and then back translated to ensure conceptual accuracy. The structured questionnaire consisted of approximately 100–200 items assessing socio-demographics, sexual behaviour, HIV/STI knowledge, service utilization, and substance use among others. The interview took approximately 30–45 min to complete. For ethical and data quality reasons, individuals who were visibly intoxicated or impaired-as assessed by trained recruiters-were not invited to participate and were offered information about the survey to return at another time. All men gave a finger-prick blood sample for HIV testing. To allay concerns regarding privacy and confidentiality, HIV tests were performed privately and anonymously at the venues by trained research nurses with valid Good Clinical Practice certificates, and participants received their results immediately after testing.

Each participant received USD 2 for their time. Men newly diagnosed with HIV were referred to key population (KP) clinics or initiation sites within their districts for antiretroviral therapy (ART). They received a referral slip to present at these sites, and research teams coordinated with facility personnel to ensure prompt ART initiation. Viral load tests were conducted on HIV-positive individuals using Dried Blood Samples (DBS), which were stored and shipped in zip lock bags with desiccation packets [33]. Participants were later contacted to obtain their viral load results at KP clinics, with participants indicating the specific clinics where they wanted to access their results. Questionnaire data included demographic characteristics, venue-going behaviour, sexual behaviour, condom use, and uptake of HIV prevention and care services. *Other variables included the Alcohol Use Disorders Identification Test (AUDIT)* [34], *a screening tool used to screen for alcohol misuse*,* and the Shona Symptom Questionnaire-14 (SSQ-14)* [35], *a validated 14-item questionnaire designed to assess psychological distress and symptoms related to common mental disorders (CMD).* The SSQ detects symptoms across seven common mental disorders categories: [1] anxiety disorders [2], depression [3], bipolar disorder [4], Post-Traumatic Stress Disorder [5], schizophrenia [6], eating disorders, and [7] disruptive behaviour/dissocial disorders [36].

### Data Management

All HIV results and questionnaire responses were entered into a database and linked using unique participant identifiers. Data from all sites were pooled for analysis. Pooling ensured greater statistical power due to increased sample size and resource optimization by conducting a single analysis compared to site-specific analysis.

### Measures

HIV testing was conducted to determine the prevalence of the infection. Viral load (VL) testing was performed using the Abbott RealTime HIV-1 viral load assay (Abbott Molecular, Des Plaines, IL, USA) to test the two whole blood spots extracted from each card and eluted in RNA lysis buffer (Promega Corporation, Madison, WI, USA). Undetectable viral load (VL), that is, virological suppression, was defined as a VL of < 1000 copies per millilitre. For mental health (SSQ-14), a score of 9 and above indicated symptoms of common mental disorders, and for alcohol use (AUDIT), a score of 8 or more was associated with harmful or hazardous drinking, and 15 or more likely indicated alcohol dependence.

Consistent condom use was defined as using condoms every time with all sexual partners in the past month. Other variables included in the analysis comprised demographic variables (survey site: Bulawayo, Gweru, Beitbridge, Harare; age: <30 years, 30–39 years, ≥ 40 years; marital status: married/living with partner, separated/divorced/widowed, never married; education: none/primary, secondary; circumcision: yes/no), behavioural variables (non-prescriptive drug use: yes/no; sex payment status: paid for sex/did not pay; STI symptoms: yes/no), and health outcome variables (HIV status: negative/positive).

We assessed non-prescription drug use (recreational/problematic controlled substances, excluding over-the-counter medications, herbal remedies, and unprescribed family/friend medications) using the World Health Organization (WHO) Alcohol, Smoking and Substance Involvement Screening Test (ASSIST) [37], a validated tool to screen for all levels of problematic substance use across 10 substance categories: tobacco, alcohol, cannabis, cocaine, amphetamine-type stimulants (ecstasy), inhalants, sedatives, hallucinogens, opioids, and “other drugs”.

### Data Weighting

Using Stata 17.0, we used a three-stage weighting scheme to achieve population representativeness: [1] Venue weights were the reverse of selection probability (total venues/sampled venues); [2] Patron/worker weights were by venue-specific sampling (total patrons/sampled patrons; venues with < 10 workers were assigned weight = 1 since they were enumerated entirely); [3] Attendance weights that adjusted for frequency of venue: respondents were assigned monthly “busy days” (Fri-Sun) based on visit frequencies (daily/4–6 times/week = 12; 2–3 times/week = 10; weekly = 4; 2–3 times/month = 2.5; ≤monthly = 1), with attendance weight = 12 / assigned days. Final weight was the product of these three weights, standardized by dividing by the mean weight to remove selection bias, preserve standard errors, and produce statistical validity. These weights were used to obtain estimates representative of men attending the sampled venues, not of the national male population.

### Statistical Analysis

Our analysis proceeded in five consecutive steps: [1] descriptive characterisation of all social venue-going men; [2] stratification of characteristics by self-reported paid sex during the past 12 months; [3–5] weighted (by PLACE design) adjusted modified Poisson models with robust standard errors examining factors associated with three outcomes—HIV status, virological suppression among HIV + men, and consistent condom use, with paid sex as the primary exposure in all models. For HIV status, we adjusted for sex payment status, site (fixed effect), age, marital status, education level, and circumcision status as confounding variables. We used available-case analysis, whereby all observations with non-missing data for the variables involved in each analysis were included. All participants accepted HIV testing, except those already aware of their HIV-positive status.

Variables such as current STI symptoms were excluded from the model because we considered them potential mediators on the causal pathway between behaviours commonly associated with paid sex (for example, multiple partners and inconsistent condom use) and HIV acquisition, rather than confounders of the relationship between reporting paid sex and HIV status. However, the directionality between paying for sex and variables such as common mental disorders symptoms, drug use, and hazardous alcohol consumption is less certain. These may either precede or co-occur with the exposure due to shared social or behavioural determinants, therefore, we excluded them from the adjusted models.

For virological suppression, we adjusted for sex payment status, site, age, marital status, and education level, but not AUDIT/ common mental disorders/drug use as potential adherence mediators and STI symptoms/condom use as biologically unrelated. For condom use, we adjusted for sex payment status, site, age, marital status, education level, and circumcision status. We excluded HIV status, STI symptoms, drug use, common mental disorders, and AUDIT because they might mediate the relationship between paying for sex and condom use or be influenced by paying for sex, acknowledging the complex/bidirectional relationships some (e.g., drug use, HIV status) may have with it. This approach allowed us to isolate the relationship between paying for sex and HIV-related outcomes while minimizing overadjustment bias caused by potential mediators in the model. All models used the first category as reference, except venue sites where the largest subgroup served as reference to ensure stable baselines and model convergence. We reported Wald test p-values. We used modified Poisson regression models to obtain prevalence ratios, which are more intuitive to interpret than odds ratios.

### Ethics Statement

This study was approved by the ethics committees at the Medical Research Council of Zimbabwe (MRCZ; MRCZ/A/2867) and the Research Council of Zimbabwe (RCZ). Participants provided informed consent by marking an “X” to confirm that the study’s nature, purpose, benefits, and risks were explained, they received HIV counselling, had time for questions, agreed to testing and discussions, and understood confidentiality. Only the study staff and a witness signed and dated the form; participants did not sign themselves.

## Results

Table 1 presents the sociodemographic, health, and behavioural characteristics of all social venue-going men. The largest proportion of participants was from Harare (62.7%), with the smallest from Gweru (11.9%). Of the men, 39.5% were aged under 30 years. Most had at least a secondary education (90.7%), and more than half were married or living with a partner (52.0%). Behaviourally, 28.2% scored ≥ 9/14 on SSQ-14 (common mental disorder symptoms), 68.3% reported non-prescriptive drug use, 27.0% met alcohol dependence criteria, and 15.2% exhibited hazardous alcohol use. Most of the men (62.6%) were not circumcised, and 40.1% reported paying for sex in the previous 12 months. Based on study HIV testing, 10.7% of the men were living with HIV, and among these men, 67.9% had achieved viral suppression. Inconsistent condom use was reported by 84.9% of men. A total of 38 out of 190 venues (20.0%) had at least half of the participants self-reporting as having paid for sex in the past 12 months.

**Table 1:** Socio-demographic, health and behavioural characteristics of all social venue-going men – (*N* = 2827)

Variable	*n*	Weighted column %
Survey site		
Bulawayo	791	13.7
Gweru	475	11.9
Beitbridge	423	12.2
Harare	1138	62.7
Age		
< 30 years	1119	39.5
30 to 39 years	918	32.9
>/= 40 years	790	27.6
Marital status		
Married/Living with Partner	1379	52.0
Separated/divorced/widowed	462	15.5
Never married	986	32.5
Education		
None or primary level	298	9.3
Secondary level	2529	90.7
Non-prescriptive drug use		
Taken any one of the non-prescriptive drugs	1904	68.3
Never took any non-prescriptive drugs	923	31.7
SSQ-14 mental health		
No symptoms of common mental disorders	2396	71.8
Symptoms suggestive of common mental disorders	431	28.2
Alcohol use (AUDIT)		
No harmful drinking	1533	57.8
Hazardous or harmful drinking	535	15.2
Alcohol dependence	759	27.0
Circumcised*		
No	1775	62.6
Yes	1033	37.4
Sex payment status		
Paid for sex	984	40.2
Did not pay for sex	1843	59.8
STI symptoms*		
Yes	217	12.8
No	2585	87.2
HIV status*		
Negative	2475	89.3
Positive	332	10.7
Condom use		
Consistent	531	15.1
Inconsistent	2295	84.9
Virological suppression among men living with HIV		
Unsuppressed	78	32.1
Suppressed	251	67.9

*Missing information: Circumcision = 19, STI symptoms = 25, HIV status = 20

Table 2 presents the distribution of venue-going men who self-reported paying for sex in the past 12 months, stratified by sociodemographic, health, and behavioural characteristics. The weighted percentage of men who self-reported paying for sex was similar in all sites (37.3% − 44.5%) except in Bulawayo (20.0%), F (1.36, 256.99) = 4.41, *p* = 0.025, where it was lower. Men who were separated, divorced, or widowed (61.8%) were more likely to report paying for sex compared to other marital groups, F (1.53, 289.88) = 9.13, *p* < 0.001. Paid sex was more common among people with only primary or no education (55.2%) than among those with secondary education (36.8%), F (1,189) = 4.90, *p* = 0.028. Men who reported alcohol dependence (59.7%), F (1, 189) = 7.05, *p* < 0.001, had STI symptoms (55.7%), F (1, 189) = 5.80, *p* = 0.017, or had symptoms of common mental disorders (58.7%), F (1, 189) = 22.12, *p* < 0.001 were likewise more likely to pay for sex. Regarding HIV status, 44.8% of men living with HIV and 44.4% of those with suppressed viral load reported paying for sex, compared to 39.6% of HIV-negative men and 46.7% of those with unsuppressed viral load, F (1.98, 374.83) = 1.17, *p* = 0.312 and F (1, 119) = 0.11, *p* = 0.743 respectively. Among those who used condoms consistently, 37.2% paid for sex compared to 40.7% who used condoms inconsistently, F (1, 189) = 0.77, *p* = 0.383.

**Table 2:** Distribution of venue-going men who self-reported paying for sex in the past 12 months, stratified by sociodemographic, health, and behavioural characteristics (*N* = 2827)

Variable	Total	*n*	Weighted row (%)	Design-Based F-test F (df1, df2)	*P*-value
Survey site					
Bulawayo	791	162	20.0	F (1.36, 256.99) = 4.41	**0.025**
Gweru	475	162	37.3		
Beitbridge	423	201	43.6		
Harare	1138	459	44.5		
Age					
< 30 years (ref)	1119	383	38.1	F (1.29, 243.32) = 1.43	0.240
30 to 39 years	918	358	44.8		
>/= 40 years	790	243	37.6		
Marital status					
Married/Living with Partner	1379	417	36.1	F (1.53, 289.88) = 9.13	**< 0.001**
Separated/divorced/widowed	462	234	61.8		
Never married	986	333	36.5		
Education					
None or primary level	298	118	55.2	F (1,189) = 4.90	**0.028**
Secondary level	2529	866	36.8		
Non-prescriptive drug use					
Taken any one of the non-prescriptive drugs	1904	568	35.7	F (1, 189) = 7.05	**< 0.001**
Never took any non-prescriptive drugs	923	416	49.8		
SSQ-14 mental health					
No symptoms of common mental disorders	2396	797	32.9	F (1, 189) = 22.12	**< 0.001**
Symptoms suggestive of common mental disorders	431	187	58.7		
Alcohol use (AUDIT)					
No harmful drinking	1533	418	30.8	F (1, 189) = 7.05	**< 0.001**
Hazardous or harmful drinking	535	225	41.0		
Alcohol dependence	759	341	59.7		
Circumcised*					
No	1775	625	42.1	F (1, 189) = 1.07	0.303
Yes	1033	356	37.4		
STI symptoms*					
Yes	217	108	55.7	F (1, 189) = 5.80	**0.017**
No	2585	869	38.0		
HIV status*					
Negative	2475	835	39.6	F (1.98, 374.83) = 1.17	0.312
Positive	332	143	44.8		
Condom use					
Consistent	531	168	37.2	F (1, 189) = 0.77	0.383
Inconsistent	2295	816	40.7		
Virological suppression					
Unsuppressed	78	35	46.7	F (1, 119) = 0.11	0.743
Suppressed	251	109	44.4		

*Missing information: Circumcision = 19, STI symptoms = 25, HIV status = 20

Factors associated with HIV status are shown in Table 3. Notably, paying for sex in the past 12 months was not significantly associated with HIV status (aPR = 1.12, 95% CI: 0.64–1.94), indicating no clear evidence of a difference in HIV prevalence between men who reported paying for sex and those who did not. The analysis revealed that age was a strong predictor of HIV status, with significantly higher prevalence among men 30–39 years (aPR = 2.37, 95% CI: 1.36–4.14) and marginally lower prevalence among those aged ≥ 40 (aPR = 4.54, 95% CI: 2.38–8.67), compared to those aged < 30 years. Marital status was also significantly associated with HIV status: separated/divorced/widowed men were more likely to be living with HIV compared to married men (aPR = 1.87, 95% CI: 1.30–2.67), while there was no significant difference for never-married men. Circumcision was significantly protective, with circumcised men being less likely to be living with HIV (aPR = 0.42, 95% CI: 0.20–0.88).

**Table 3:** Factors associated with HIV status among men tested for HIV at venues (*N* = 2807)

Explanatory variables	Number (*n*)	Number of social venue-going men who are living with HIV: *N* = 332	Unweighted row %	PLACE-design weighted row %	Unadjusted Prevalence Ratios (95% CI)	Adjusted Prevalence Ratios (95% CI)	Adjusted Wald test *P*-value
Sex payment status in the past 12 months							
Did not pay for sex	1829	189	10.3	9.9	1	1	0.693
Paid for sex	978	143	14.6	11.9	1.21 (0.70–2.08)	1.12 (0.64–1.94)	
Survey sites							
Bulawayo	791	112	14.2	11.9	0.72 (0.50–1.05)	0.83 (0.56–1.22)	
Gweru	475	44	9.3	10.7	0.65 (0.42–1.02)	0.80 (0.51–1.27)	
Beitbridge	423	69	16.3	16.5	1	1	0.454
Harare	1118	107	9.6	9.3	0.56 (0.32–0.98)	0.67 (0.44–1.04)	
Age							
< 30 years	1109	34	3.1	4.5	1	1	**< 0.001**
30 to 39 years	912	101	11.1	10.3	2.30 (0.85–6.24)	2.37 (1.36–4.14)	
>/= 40 years	786	197	25.1	20.1	4.52 (1.79–11.41)	4.54 (2.38–8.67)	
Marital status							
Married/living with a partner	1369	171	12.5	10.5	1	1	**0.047**
Separated/divorced/widowed	460	105	22.8	20.2	1.92 (1.07–3.46)	1.87 (1.30–2.67)	
Never married	978	56	5.7	6.5	0.62 (0.28–1.37)	1.60 (0.95–2.69)	
Level of education							
None or primary	296	54	18.2	17.4	1	1	0.125
Secondary or higher	2511	278	11.1	10.0	0.58 (0.34–0.96)	0.68 (0.40–1.18)	
Circumcision status*							
Uncircumcised	216	41	19.0	23.9	1	1	**0.001**
Circumcised	2566	286	11.2	8.7	0.33 (0.21–0.51)	0.42 (0.20–0.88)	

Adjusted for site, age, marital status, education level, sex payment status, and circumcision status

p-value is from the Wald test,

* Missing information: Circumcision = 19

Factors associated with virological suppression are shown in Table 4. Paying for sex in the past 12 months was not significantly associated with virological suppression (aPR = 0.97, 95% CI: 0.71–1.34). Among 251 men living with HIV, the highest viral suppression levels were in Bulawayo (80.4%), while Harare had the lowest (59.8%, aPR = 0.76, 95% CI: 0.52–1.11), though this difference was not statistically significant. Suppression did not significantly vary by age, with men aged ≥ 40 years having no different suppression from those < 30 years (aPR = 1.02, 95% CI: 0.66–1.56). Marital status, education, and payment status for sex also did not have any significant associations with virological suppression. No predictors of virological suppression were identified in the adjusted models overall.

**Table 4:** Factors associated with virological suppression among HIV-positive men attending social venues - (*N* = 329)

Explanatory variables	Number (*n*)	Number of social venue-going men who are virologically suppressed: *N* = 251	Unweighted row %	PLACE-design weighted row %	Unadjusted Prevalence Ratios (95% CI)	Adjusted Prevalence Ratios (95% CI)	Adjusted Wald test *P*-value
Sex payment status in the past 12 months							
Did not pay for sex	185	142	76.8	68.9	1	1	0.864
Paid for sex	144	109	75.7	66.7	0.97 (0.61–1.53)	0.97 (0.71–1.34)	
Survey site							
Bulawayo	109	90	82.6	80.4	1	1	0.518
Gweru	44	35	79.6	79.2	0.98 (0.78–1.24)	0.94 (0.75–1.18)	
Beitbridge	69	49	71.0	73.8	0.92 (0.74–1.13)	0.90 (0.69–1.17)	
Harare	107	77	72.0	59.8	0.74 (0.47–1.17)	0.76 (0.52–1.11)	
Age							
< 30 years	35	20	57.1	76.8	1	1	0.347
30 to 39 years	98	71	72.5	51.8	0.67 (0.37–1.22)	0.71 (0.41–1.21)	
≥ 40 years	196	160	81.6	74.5	0.97 (0.64–1.46)	1.02 (0.66–1.56)	
Marital status							
Married/living together	167	134	80.2	71.0	1	1	0.683
Separated/widowed/divorced	107	81	75.7	57.5	0.81 (0.45–1.46)	0.82 (0.52–1.31)	
Never married	55	36	65.5	76.0	1.07 (0.71–1.60)	1.02 (0.71–1.46)	
Level of education							
None or primary level	53	38	71.7	78.0	1	1	0.314
Secondary or higher	276	213	77.2	66.2	0.85 (0.62–1.17)	0.88 (0.68–1.13)	

Adjusted for site, age, marital status, education level, and sex payment status

All percentages presented in this table are weighted using PLACE-design weights to account for differential selection probabilities across sites and strata

p-value is from the Wald test

Factors associated with reporting consistent condom use in the previous month are shown in Table 5. Our findings demonstrate that consistent condom use in the previous month among men who frequented social venues was comparatively low across all sites. In adjusted analysis, notably, paying for sex in the past 12 months was not significantly associated with consistent condom use (aPR = 0.88, 95% CI: 0.60–1.31). Men in Harare were significantly less likely than those in Beitbridge to use condoms consistently (aPR = 0.58, 95% CI: 0.39–0.87). Marital status was strongly associated with condom use: never-married men were considerably more likely to use condoms consistently than married men (aPR = 3.59, 95% CI: 2.41–5.34). There was no evidence that men with secondary or higher education level (aPR = 1.41, 95% CI: 0.96–2.08) were more likely to report consistent condom use compared with men with none or primary education level.

**Table 5:** Factors associated with consistent condom use among all venue-going men - (*N* = 2,827)

Explanatory variables	Number (*n*)	Number of social venue-going men who self-reported consistent condom use in the past month: *N* = 561	Unweighted row %	PLACE-design weighted row %	Unadjusted Prevalence Ratios (95% CI)	Adjusted Prevalence Ratios (95% CI)	Adjusted Wald test *P*-value
Sex payment status in the past 12 months							
Did not pay for sex	1843	363	19.7	15.8	1	1	0.522
Paid for sex	984	168	17.1	14.0	0.88 (0.60–1.31)	0.87 (0.57–1.33)	
Survey sites							
Bulawayo	791	167	21.1	18.7	0.91 (0.69–1.21)	0.72 (0.51-1.00)	
Gweru	475	103	12.7	18.4	0.90 (0.66–1.21)	0.84 (0.62–1.13)	
Beitbridge	423	88	20.8	20.6	1	1	**0.041**
Harare	1138	173	15.2	12.5	0.61 (0.41–0.91)	0.58 (0.39–0.87)	
Age							
< 30 years	1119	283	25.3	20.5	1	1	0.512
30 to 39 years	918	133	14.5	10.7	0.52 (0.36–0.75)	0.94 (0.69–1.29)	
>/= 40 years	790	115	14.6	12.5	0.61 (0.36–1.04)	1.30 (0.79–2.14)	
Marital status							
Married/living with a partner	1379	104	7.5	7.7	1	1	**< 0.001**
Separated/divorced/widowed	462	130	28.1	18.0	2.35 (1.38–4.01)	2.47 (1.42–4.31)	
Never married	986	279	30.2	25.4	3.32 (2.01–5.48)	3.59 (2.41–5.34)	
Level of education							
None or primary	298	48	16.1	11.0	1	1	**0.080**
Secondary or higher	2529	483	19.1	15.5	1.41 (0.89–2.24)	1.41 (0.96–2.08)	
Circumcision status*							
Uncircumcised	1775	311	17.5	14.2	1	1	0.733
Circumcised	1033	217	21.0	16.5	1.16 (0.79–1.71)	1.06 (0.76–1.49)	

Adjusted for site, age, marital status, education level, circumcision status, and sex payment status

p-value is from the Wald test

* Missing information: Circumcision = 19

## Discussion

To better inform prevention and treatment programming among men in Zimbabwe, we assessed HIV prevalence, virological suppression, and HIV-related behaviours among men at social venues in four cities and towns. Among 2,827 venue-going PLACE men, 984 (40.1%) reported paying for sex in the previous year. HIV prevalence was 10.7% overall, and among these men living with HIV, only 68.9% were virologically suppressed; below the UNAIDS 2030 target of 86% [1]. Few men (15.1%) reported consistent condom use. We found no significant differences in HIV status, virological suppression, or consistent condom use between men who paid for sex and those who did not. The combination of high HIV prevalence, prevalent substance use, inconsistent condom use, and gaps in viral suppression underscores substantial HIV risk and high prevention and treatment needs among all venue attendees.

Among Zimbabwean social venue attendees, 40.1% reported paying for sex in the past year (with substantial site variation), exceeding rates from East African venue samples (20.0–22.5%, likely reflecting underreporting) [38], Sub-Saharan Africa (SSA) population-representative surveys (2.7% past year; 8.0% lifetime) [39], and Zimbabwe Demographic and Health Survey 2015 (4.0% past year; 18.0% lifetime—probable underestimates) [4]. The high rate of recent paid sex (49.0% in past 4 weeks) confirms ongoing high-risk exposure. While direct African venue data are scarce, studies of high-risk occupational groups (e.g., truck drivers/miners: 6.6–47.8% past year) show comparable prevalences. Many individuals from these occupational groups likely visit the venues included in the study, making them a relevant comparison group [40–44]. However, other studies of high-risk subgroups report lower proportions [45–49].

HIV prevalence among venue-going men aged ≥ 16 years was 10.7% in our study, with no significant difference by sex payment status. This aligns with Zimbabwe national surveys: Zimbabwe Population-Based HIV Impact Assessment (ZIMPHIA) 2020 (10.2% men 15–49 years) [50], and Zimbabwe Demographic and Health Survey 2015 (10.5% men ≥ 15 years) [4]. All three sources show higher prevalence among men ≥ 35 years. Contrary to our hypothesis, we found no independent association between reporting paid sex and HIV status. This suggests that in this population, HIV risk may be driven by the specific high-risk behaviours (e.g., inconsistent condom use, multiple partnerships). Notably, Zimbabwe Demographic and Health Survey 2015 found men reporting paid sex were more likely to be living with HIV than non-payers. While contextual differences limit direct comparison, our prevalence was lower than among South African venue men (15.5%) [10].

Paid sex-associated risk factors (multiple partners, condom negotiation difficulties) increase HIV acquisition risk, particularly given persistently higher HIV prevalence among female sex workers despite improved ART access. Unusually, venue-going men showed HIV prevalence comparable to general population males (10.2%), suggesting potential protective partner selection or behaviours. These findings confirm that venue-going men constitute a significant proportion of people living with HIV, necessitating targeted interventions.

Virological suppression among men living with HIV was nominally lower in our study (68.9%) than in Zimbabwe Population-Based HIV Impact Assessment 2020 (73.0% among men ≥ 15 years) [50]. This gap may reflect poorer ART engagement in high-risk venue-going populations and contextual/demographic differences. Both rates fall substantially below UNAIDS’ 86.0% suppression target, representing the proportion of all people living with HIV who are virologically suppressed if the targets are met [1].

Consistent condom use was low: only 15.1% overall and 14.0% of recent sex-payers self-reported consistent use in the past month. While inconsistent use is commonly documented across populations [40–43, 47, 51–54], direct comparison with Zimbabwe Demographic and Health Survey 2015 (90.2% condom use at last paid sex among payers) [4] is limited by our measure’s lack of partner-type distinction. Other high-risk groups show similar gaps: 34.8% of truck drivers reported consistent use (past 3 months) [51], and 35.7% used condoms at last vaginal sex in East African venues in past year [29]. Despite generally high condom access, these patterns underscore widespread unsafe sex among venue-going men, elevating HIV transmission risk.

Despite venue-going men exhibiting higher overall HIV risk and poorer prevention/care engagement than the general population, we observed no elevated HIV prevalence among those reporting paid sex versus non-payers. This apparent anomaly may stem from underreporting of paid sex by men living with HIV due to stigma/social desirability bias or genuine behavioural differences (e.g., reduced sexual activity). This anomaly may also arise due to comparable risk behaviours among non-payers (e.g., multiple/casual partnerships) diminishing observable prevalence differences.

Our venue-based surveys effectively reached men who pay for sex, a group often missed in traditional surveys. We argue that conducting surveys during peak times improved efficiency, engaging local stakeholders fostered community acceptance, and probability sampling enhanced representativeness and generalizability to venue-going men. However, our study did have a number of limitations. Self-reported data could have resulted in social desirability/recall bias [55–59]. The cross-sectional design could prevent causal/temporal inferences, and demographic/cultural/socioeconomic/healthcare variations, and non-random province selection may constrain the generalizability of our results. In addition, our small virological suppression sample and our inability to distinguish condom use by partner type (spouse vs. others) for married men limit robustness. Future research should differentiate condom use by partner.

We applied sampling weights in PLACE surveys. Larger discrepancies between weighted and unweighted estimates occurred for venue-clustered variables (age, education, drug use, paid sex status), where weighting amplifies/reduces the influence of venues with distinct characteristics. Paid sex showed strong behavioural clustering: 20.0% of venues contained > 50.0% of men reporting it, with methodological (weighting), epidemiological (hotspot targeting), and programmatic (resource allocation) implications. Subgroups over-/under-sampled relative to the broader population received heavier/lighter weights, while evenly distributed variables required smaller adjustments.

These findings demonstrate that all venue-going men—not only those reporting paid sex—exhibit substantial HIV prevention and treatment gaps warranting targeted intervention, giving rise to several priority recommendations. Routine mental health and substance use screening should be integrated into HIV services targeting venue-going men, given the high co-occurrence of these conditions (substance use 68.3%, hazardous alcohol use 42.0%, mental distress 28.2%) that undermine HIV prevention and care continuum outcomes. Secondly, the gap in the treatment cascade needs to be filled by the application of low‑barrier, community‑centric strategies, such as community distribution of ART (PODIs), refill groups (CARGs), peer-led adherence clubs, and mHealth assistance, to address the huge gap in the rates of achieved virality (a meagre 67.9% among men living with HIV) [60–64]. Thirdly, prevention needs to be escalated and updated, particularly where the risks are highest. This requires: [1] discreet condom availability in venues [2], multi-location HIV self-testing kits, and [3] oral/long-acting PrEP (CAB-LA)—targeting men with persistent high-risk HIV-endemic behaviours [65, 66]. Additionally, prioritizing treatment as prevention ensures early ART initiation and sustained care for men living with HIV.

## Conclusion

Outcomes from our study indicate that there are unmet service needs in terms of HIV prevention and treatment among social venue-going men, as there are gaps in the care continuum and associated risk factors. There is obvious unmet treatment need, considering that only 67.9% of the men living with HIV had suppressed viral loads, even though 10.7% had been diagnosed. There is a lack of effective measures for the prevention of HIV infections, considering the high prevalence of inconsistent condom use, drug and common mental health issues among the target population. There is a clear need for integrated services that address substance use, mental health, and HIV together, to reduce transmission and improve health outcomes.

Venue sampling proved feasible for reaching the diverse, hard-to-reach population of men having sex with FSWs. These findings address the evidence gap on HIV in African venue-going men. Future interventions should explore on-site service delivery at social venues to reduce HIV risk, while longitudinal studies could monitor changes in prevalence, adherence, and sexual behaviours.

## Data Availability

The data that supports the findings of this study are openly available in: Open Science Framework: https://osf.io/rsd9z/files/osfstorage.
